# A Purinergic P2 Receptor Family-Mediated Increase in Thrombospondin-1 Bolsters Synaptic Density and Epileptic Seizure Activity in the Amygdala-Kindling Rat Model

**DOI:** 10.3389/fncel.2018.00302

**Published:** 2018-10-01

**Authors:** Hongliu Sun, Luyu Ma, Yurong Zhang, Xiaohong Pan, Chaoyun Wang, Jinjin Zhang, Xiuli Zhang, Hongwei Sun, Qiaoyun Wang, Wei Zhu

**Affiliations:** ^1^School of Pharmaceutical Sciences, Binzhou Medical University Yantai, China; ^2^Shandong Academy of Medical Sciences (SDAMS) Jinan, China

**Keywords:** thrombospondin-1, purinergic receptor 2, synapse, epileptogenesis, astrocyte

## Abstract

Previous studies suggested that the thrombospondin-1/transforming growth factor-β1 (TSP-1/TGF-β1) pathway might be critical in synaptogenesis during development and that the purinergic P2 receptor family could regulate synaptogenesis by modulating TSP-1 signaling. However, it is unclear whether this pathway plays a role in synaptogenesis during epileptic progression. This study was designed to investigate this question by analyzing the dynamic changes and effects of TSP-1 levels on the density of synaptic markers that are related to epileptic seizure activity. In addition, we evaluated whether P2-type receptors could regulate these effects. We generated a rat seizure model via amygdala kindling and inhibited TSP-1 activity using small interfering RNA (siRNA) interference and pharmacological inhibition. We treated the rats with antagonists of P2 or P2Y receptors, pyridoxalphosphate-6-azophenyl-2’,4’-disulfonic (PPADS) or Reactive Blue 2. Following this, we quantified TSP-1 and TGF-β1 immunoreactivity (IR), the density of synaptic markers, and seizure activity. There were significantly more synapses/excitatory synapses in several brain regions, such as the hippocampus, which were associated with progressing epileptic discharges after kindling. These were associated with increased TSP-1 and TGF-β1-IR. Genetic or pharmacologic inhibition of TSP-1 significantly reduced the density of synaptic/excitatory synaptic markers and inhibited the generalization of focal epilepsy. The administration of PPADS or Reactive Blue 2 attenuated the increase in TSP-1-IR and the increase in the density of synaptic markers that follows kindling and abolished most of the epileptic seizure activity. Altogether, our results indicate that the TSP-1/TGF-β1 pathway and its regulation by P2, particularly P2Y-type receptors, may be a critical promoter of synaptogenesis during the progression of epilepsy. Therefore, components of this pathway may be targets for novel antiepileptic drug development.

## Introduction

Epilepsy is considered a network-level disease, where abnormal, synchronized firing of a population of neurons in one location spreads outward via synaptic connections to involve other areas of the brain, which in turn spread the wave further. The neural network mediates this propagation from focal to generalized seizure activity (Haneef et al., 2015; Xiao et al., 2015; Kros et al., 2017; Sánchez-Ramón and Faure, 2017). Epileptic activity can induce excitotoxic damage locally, but it can also propagate through white matter tracts and damage distant areas, including areas that are contralateral to the focus (Feldt Muldoon et al., 2013; Burns et al., 2014; Caciagli et al., 2014; Xu et al., 2014). For example, complex partial seizures in patients with epilepsy were found to begin at abnormal hippocampal synapses in the middle temporal lobe (Colder et al., 1996) and were found to propagate through the hippocampal network to cause temporal lobe epilepsy (Garcia-Ramos et al., 2017; Lupica et al., 2017). This propagation can cause a pathological restructuring of the affected networks by increasing synaptogenesis that facilitates the development and spread of seizure activity (Fidzinski et al., 2015; Heller and Rusakov, 2015; Amakhin et al., 2016). In fact, in the normal brain, changing the synaptic number, connection strength, and local network connectivity can induce epileptiform activity (Netoff et al., 2004). Consequently, interfering with this synaptogenesis may inhibit, or at least delay, the formation of the epileptic network.

In recent years, astrocytes have been found to function not only as neuronal support cells and neuroimmune cells but also as regulators that stabilize synapses (Codazzi et al., 2015; Murphy-Royal et al., 2015; Charvériat et al., 2017; van Deijk et al., 2017; Dubový et al., 2018; Milton and Smith, 2018). Barres and Smith (2001) found that including astrocytes in neuronal cultures, even as a feeder lay, can significantly increase synapse formation, and these synapses demonstrated normal postsynaptic potentials and frequency multiplication (Ullian et al., 2001). By contrast, purified neuronal cultures demonstrated only minimal synapse formation, indicating that astrocytes may secrete one or more extracellular signal molecules that strongly support synaptogenesis (Ullian et al., 2001).

One such molecule that is secreted by astrocytes is the extracellular matrix protein, thrombospondin-1 (TSP-1). In normal mouse brain development, TSP-1 localization and timing were correlated with synaptogenesis, and the TSP-1 knockout significantly reduced the number of new synapses (Pfieger and Barres, 1997). Another study confirmed that adding either purified TSP-1 or astrocytes to neuronal cultures generated the same synaptic microstructure and vesicle number in both the culture conditions (Christopherson et al., 2005). These studies strongly indicate that TSP-1 produced by astrocytes is a key molecule in synapse formation. Upstream of TSP-1, TSP-1 expression and secretion are positively regulated by astrocytic purinergic P2-type (extracellular ATP-binding) receptors. For example, administration of a P2 family receptor antagonist decreases the expression of TSP-1 by 80% (Ribeiro et al., 1999). Further research demonstrated that the P2Y4 subtype (Tran et al., 2012), which is coupled to protein kinase signaling pathways that include p38/MAPK and Akt, is the primary mechanism through which TSP-1 is regulated (Diniz et al., 2012). Downstream of TSP-1, TSP-1 activates latent transforming growth factor-β1 (TGF-β1), which, according to evidence, is an important effector of TSP-1-mediated synaptogenesis. Activated TGF-β1 that was added to cell culture media significantly increased the number of synapses, with normal ultrastructural and electrophysiological features (Tran and Neary, 2006). TSP-1 was found to activate TGF-β1 by binding the Leu-Ser-Lys-Leu (LSKL) peptide sequence in the latency-associated peptide (LAP) region of the latent TGF-β1 complex (Tran et al., 2012). Notably, administration of exogenous LSKL peptide can block binding by TSP-1, significantly inhibiting the activation of the molecule (Tran et al., 2012).

These data indicate that the P2-type receptor-regulated TSP-1/TGF-β1 pathway is highly active in developmental synaptogenesis. Given the similar mechanisms of synaptogenesis due to epileptic activity, it can be hypothesized that this pathway may also be involved in synaptogenesis that is related to epileptic activity or its propagation. This study was designed to test this hypothesis in a rat model of epilepsy in which focal kindling of the amygdala had progressed to generalized seizure activity. We quantified the immunoreactivity (IR) of TSP-1 and synaptic number in Sprague-Dawley model rats, and we compared these data to model rats administered with TSP-1 small interfering RNA (siRNA) or LSKL peptide (a TSP-1 antagonist), the broad-acting P2 receptor antagonist pyridoxalphosphate-6-azophenyl-2’,4’-disulfonic acid (PPADS), or the P2Y receptor antagonist Reactive Blue 2. We also analyzed epileptiform activity in the rat groups to identify associations between TSP-1 density and epileptiform activity in the kindled rat model.

## Materials and Methods

### Animals and Surgery

We used male Sprague-Dawley rats (Certificate No. SCXK2014-0006; provided by Jinan Jinfeng Experimental Animal Co. Ltd, Shandong, China), weighing 280–300 g, that were fed separately and given water and food *ad libitum*. All experiments were conducted in accordance with the ethical guidelines of the Binzhou Medical University Animal Experimentation Committee (approval no. 2015005) and in complete compliance with the National Institutes of Health (NIH) *Guide for the Care and Use of Laboratory Animals* (NIH Publications No. 8023, revised 1996). The experiments were performed between 9:00 and 17:00. All efforts were made to minimize the number of animals used and their suffering. A total of 286 rats were used in this study.

Rats were mounted on a stereotactic apparatus after anesthesia (chloral hydrate, 400 mg/kg, intraperitoneal injection). Electrodes (diameter 0.2 mm, A.M. Systems, Sequim, WA, USA) were made of teflon-coated, twisted stainless steel wires with an uncoated tip that was 0.5 mm long and were implanted into the right basolateral amygdala (anteroposterior: −2.4 mm, lateral: −4.8 mm, ventral: −8.8 mm). Kindling stimulation and electroencephalograms (EEGs) were recorded through the same electrodes by using a PowerLab system (ADInstruments, Sydney, NSW, Australia). A stainless steel cannula (Reward, China) was implanted into the right lateral cerebral ventricle (anteroposterior: −1.8 mm, lateral: −0.96 mm and ventral: −3.8 mm) as previously described (Sun et al., 2016). Animals were allowed to recover from surgery for 10 days.

### Kindling and Epileptic Seizure Analysis

Stimulation of the amygdala (1 s, monophasic square-wave pulses) were delivered at 60 Hz, while EEG monitoring and recording were carried out from about 5 min before kindling stimulation through to the cessation of afterdischarge (AD). The AD threshold (ADT) for each animal was determined on day 0 as previously reported (Sun et al., 2017). All animals were subsequently subjected to kindling stimulation of the same current intensity as the determined ADT once daily, and the seizure stage (see below) and AD duration (ADD) were recorded for 15 min. Control rats were connected to the stimulator for 15 min, but no current was delivered.

Epileptic seizure severity during kindling progression was staged as 1–5 using Racine’s criteria (Sun et al., 2017). In short, stages 1–3 were considered focal seizures, while stages 4 and 5 were considered generalized seizures. The ADT was determined after the last stimulation at the end of behavioral testing. Electrode placements were histologically verified. Only animals with electrodes that were correctly implanted in the basolateral amygdala and with successful right lateral cerebral ventricle cannulation were included in the statistical analysis.

### Drug Administration and siRNA Interference

After determining the ADT, either LSKL (50, 100, or 200 μg, Sigma, St. Louis, MO, USA), PPADS (10, 20, or 30 μg, Abcam, Cambridge, MA, USA), Reactive Blue 2 (20 μg, Bomei, China), or saline in 5 μl volume was injected once daily into the right lateral cerebral ventricle, over a period of 10 min, using a disposable dental needle. The needle was held in place for 5 min before being slowly retracted. siRNA was designed and synthesized by Tuoran Biological Technology Co., Ltd. (Shanghai, China) using the following oligonucleotide sequences: 5’-GCCAGUAUGUUUACAACGUdTdT-3’ and 5’-ACGUUGUAAACAUACUGGCdTdT-3’. Negative controls were produced using the following oligonucleotides: 5’-UUCUCCGAACGUGUCACGUTT-3’ and 5’- ACGUGACACGUUCGGAGAATT-3’.

Increasing doses of siRNA (0.5, 1.5, 2.5 μg) or negative control (control; all 5 μl) were injected into the right lateral cerebral ventricle every other day. The injection was performed over a period of 10 min, after which the needle was held in place for 5 min. Behavioral seizure activity and EEGs in the amygdala were recorded after kindling stimulation every day. The ADT was then determined again after the stimulations.

### Immunohistochemistry

During every Racine stage (1–5) in the kindling group or on the 10th day of amygdala kindling in drug/siRNA treated groups, four rats out of each group were deeply anesthetized and perfused intracardially with 4% paraformaldehyde in PBS. Coronal slices, 10 μm thick, were prepared by using a cryostat (CM3050s, Leica, Germany). In every group, immunofluorescence staining for post synaptic density protein 95 (PSD-95, 1:200, Abcam, ab2723) and double-immunofluorescence staining for glial fibrillary acidic protein (GFAP, 1:100, Beijing Zhongshan, ZA-0117)/TSP-1 (1:100, Abcam, ab1823) were performed. Sequentially, the sections were incubated with secondary antibodies (fluorescein isothiocyanate (FITC)-conjugated, 1:200, EMD Millipore; cyanine-3 (Cy3)-conjugated, 1:200, Beyotime Institute of Biotechnology), after washing it thrice with 0.01M PBS, the sections were coverslipped and observed under a fluorescence microscope (CX41, Olympus, Japan). In addition, the optical density of IR was quantified with ImageJ 1.37 software (NIH, Bethesda, MD, USA). For additional analysis, three fields (80 μm × 60 μm/field) were selected randomly in every 200× microscope view, and PSD-95-positive puncta in the fields were counted and averaged.

### Western Blot Analysis

As previously described in the immunohistochemistry section, four rats from each group at the same time points were deeply anesthetized and decapitated, and the brains were removed without delay. The excised brains were then microdissected into the hippocampus, piriform cortex (PC), remaining cortex (except the PC), and amygdala and were individually sonicated on ice. Their protein content was quantified as previously described (Sun et al., 2013). Homogenates were mixed with sample loading buffer, separated on 12% SDS-polyacrylamide gels, and electrically transferred onto PVDF membranes. After blocking with 5% skimmed milk for 1 h, the membranes were incubated with mouse monoclonal antibody against synapsin-I (1:1,000, Abcam, ab8), PSD-95 (1:1,000; Abcam, ab2723), vesicular glutamate transporter-1 (vGluT-1, 1:1,000; Abcam, ab106289), or glyceraldehyde-3-phosphate dehydrogenase (GAPDH, 1:2,000, Goodhere, AB-P-R-001) at 4°C overnight. Immunoreactive bands were visualized by using enhanced chemiluminescence via horseradish peroxidase-conjugated IgG secondary antibodies. The normalized intensity relative to GAPDH was obtained to verify equal loading.

### Flow Cytometry

Brains were obtained from four rats per Racine stage and microdissected as previously described, and then they were rapidly soaked in cell staining buffer. Single cell suspensions were prepared by using filtration and then fixed with 4% paraformaldehyde for 10 min, followed by 0.1% triton-X 100 treatment for 10 min, and then they were washed with 0.01M PBS via repetitive centrifugation; cells were resuspended and counted with cell staining buffer. Cells were diluted to 5–10 × 10^6^ cells/ml, and 100 μl of cell suspension (5–10 × 10^5^ cells/tube) was added to each detection tube. Blocking was accomplished by treating with 5% bovine serum albumin (BSA) for 10 min in an ice bath. Monoclonal anti-TGF-β1 (1:100; R&D, Minneapolis, MA, USA, MAB240) antibody was administered into each tube, after which the tubes were incubated at 4°C for 20 min. After washing thrice with 0.01M PBS, the cells were treated with the secondary antibody, FITC-conjugated anti-mouse IgG (1:400, Biyuntian, China, A22110), for 15 min. After washing thrice with 0.01M PBA, the samples were analyzed for green (500–550 nm) fluorescence by using a flow cytometer (Becton Dickinson, Franklin Lakes, NJ, USA) with CellQuest analysis software (BD Biosciences, San Jose, CA, USA).

### Statistical Analysis

All data are presented as mean ± SEM. Statistical analysis was performed by SPSS v13.0 for Windows. After normal distribution test, one-way analysis of variance (ANOVA) and Tukey’s *t*-test were used for the normally distributed data (*P* > 0.1), and nonparametric Kruskal-Wallis H test was used for the comparison of non-normally distributed data (*P* < 0.1) in terms of IR, change of ADT, and seizure stage in every group. Analysis of group progression of seizure stage and ADD during kindling acquisition was performed by two-way ANOVA for repeated measures followed by Tukey’s *t*-test. For these analyses, *P* < 0.05 was considered significant.

## Results

### Increased Immunoreactivity of TSP-1 and Synaptic Number During Amygdala Kindling

**Figure 1:**
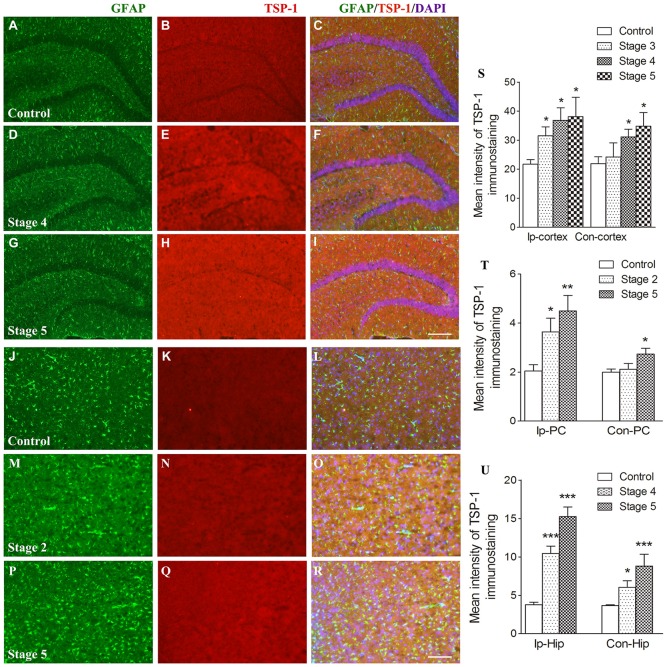
Increased immunoreactivity (IR) of thrombospondin-1 (TSP-1) in the amygdala-kindled rat model. The represented changes in IR of TSP-1 that begin at different stages in different subregions are presented. IR of glial fibrillary acidic protein (GFAP; green) and TSP-1 (red) in the ipsilateral hippocampus (**A–I**, bar = 200 μm) and ipsilateral piriform cortex (PC; **J–R**, bar = 200 μm) during seizure acquisition induced by amygdala kindling (Ip-cortex, ipsilateral cortex, except PC; Con-cortex, contralateral cortex except PC). DAPI, blue. The mean intensity of TSP-1-IR was significantly increased when compared with the controls (**S–U**, *n* = 4 per group). Data are shown as mean ± SEM. *P < 0.05, **P < 0.01 and ***P < 0.001 compared with controls.

When compared with the control group, the IR of TSP-1 after kindling increased in different brain regions progressively as epileptic activity increased (stage 1–5; Figure 1). Beginning at seizure stage 2, an increase in TSP-1-IR was observed in the ipsilateral PC (78.1% higher than the control, *P* < 0.05; Figures 1N,Q,T). Beginning at seizure stages 4 and 3, an increase in TSP-1-IR was demonstrated by the ipsilateral hippocampus and cortex (except the PC; 2.83- and 1.45-fold higher than the control, *P* < 0.001 and *P* < 0.05, respectively; Figures 1E,H,S,U). Notably, an increase in TSP-1-IR was observed in the contralateral hippocampus (*P* < 0.05; Figure 1U) and cortex (*P* < 0.05; Figure 1S), beginning at seizure stage 4. These data indicate that TSP-1-IR increased synchronously with the spread of kindling-induced seizure activity, from the kindled amygdala (data not shown) and ipsilateral PC to other ipsilateral regions, and, finally, to the contralateral hippocampus and cortex (Figure 1). No significant differences in TSP-1-IR were found in other subregions or at other seizure stages (data not shown).

**Figure 2:**
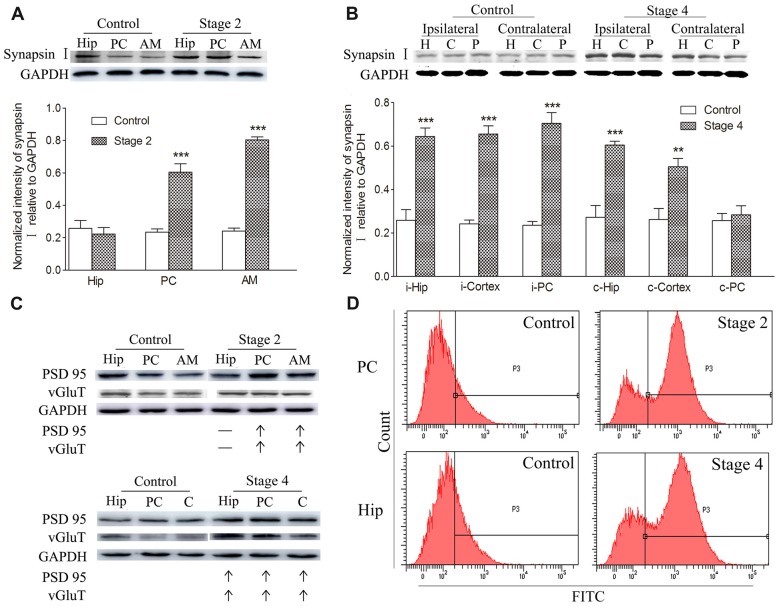
Increased synaptic density and transforming growth factor-β1 (TGF-β1) during epileptic progression. The represented changes in IR of related proteins that begin at different stages in different subregions are presented. Measurements of synapsin-I-IR in the ipsilateral hippocampus (Hip), PC and amygdala (AM) in stage 2 **(A)** and bilateral hippocampus (H), piriform cortex (P) and cortex except PC (C) in stage 4 (**B**; i-ipsilateral; c-contralateral) by western blotting during epileptic progression (*n* = 4 per group). Ipsilateral IR of post synaptic density protein-95 (PSD-95) and vesicular glutamate transporter-1 (vGluT-1) in stage 2 and 4 (**C**, no changes, –; increase, ↑; *n* = 4 per group). Flow cytometry-based quantification of TGF-β1 (**D**, *n* = 4 per group). The bands were excised from different gels, which were run under the same electrophoresis condition. Data are shown as mean ± SEM. **P < 0.01 and ***P < 0.001 compared with controls.

Synaptic density, represented by synapsin-I IR in western blots during kindling, demonstrated increases that were synchronized to those of TSP-1-IR. The IR of synapsin-I in the ipsilateral amygdala and PC were 3.33- and 2.56-fold higher than the control group, respectively, when seizures progressed to stage 2 (*P* < 0.001 and 0.001; Figure 2A). A greater density of synaptic markers was found in the remaining ipsilateral cortex (except the PC), beginning at seizure stage 3 (data not shown), and in the ipsilateral hippocampus, beginning at stage 4 (*P* < 0.001; Figure 2B). In the contralateral hippocampus and the remaining cortex, the IR of synapsin-I was 2.22- and 1.93-fold greater than those in the control group, respectively (*P* < 0.001 and *P* < 0.01; Figure 2B).

Further experiments were performed to investigate the changes in excitatory synapses. These results confirmed the increases in the excitatory postsynaptic marker, vGluT-1/PSD95, (via western blot; Figure 2C) in synchrony with the increased IR of synapsin-I and TSP-1 during the kindling progression. Additionally, a parallel change was observed in TGF-β1-IR while using flow cytometry, which was consistent with the increase in TSP-1-IR and synapse numbers during kindling (Figure 2D).

### Inhibition of TSP-1 Activity Reduced Both Synaptic Density and Evoked Seizures

**Figure 3:**
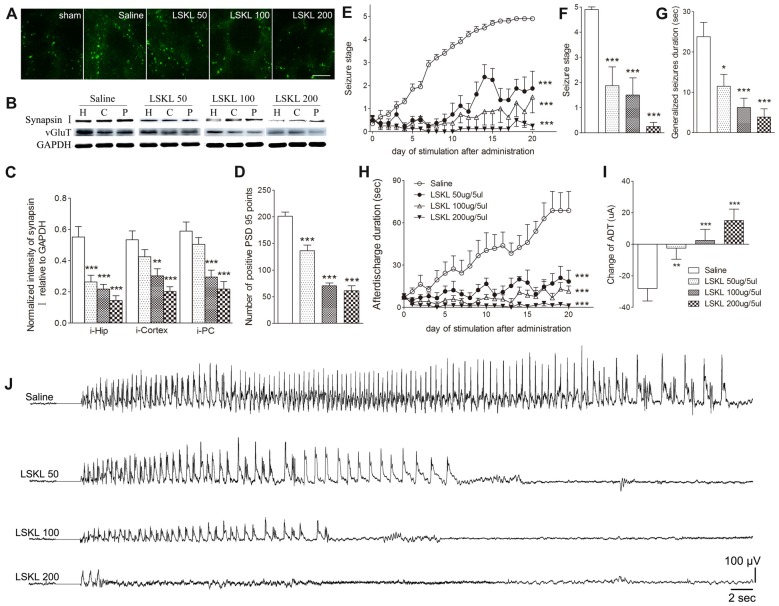
Inhibitory effects of the leu-ser-lys-leu (LSKL) peptide delivered to the lateral ventricle on synapses and kindling progression. **(A)** IR of PSD-95 (green, sham group, without kindling; bar = 10 μm), **(B,C)** the ipsilateral IR of synapsin-I/vGluT-1 after different doses of LSKL (H, hippocampus; P, piriform cortex; C, cortex except PC), **(D)** number of PSD-95-positive puncta in the field (80 μm × 60 μm), **(E)** Racine stage progression of epilepsy, **(F)** seizure stages after 20 stimulations, **(G)** generalized seizure duration after 20 stimulations, **(H)** afterdischarge duration (ADD), **(I)** changes in AD threshold (ADT) and **(J)** respective electroencephalograms (EEGs) recorded from the amygdala in kindled rats (saline group, *n* = 10; LSKL groups, *n* = 8–10 per group). The bands were excised from different gels, which were run under the same electrophoresis condition. Data are shown as mean ± SEM. Asterisks show significant differences from the control group (*P < 0.05; **P < 0.01; ***P < 0.001).

The relationship among increased TSP-1, the density of synaptic markers, and seizure progression was assessed by inhibiting TSP-1 with LSKL during kindling progression. Treatment with LSKL significantly slowed the progression of kindling (*P* < 0.001; Figure 3E) and attenuated synapsin-I-IR (*P* < 0.01 and *P* < 0.001; Figures 3B,C), PSD-95-IR (*P* < 0.001; Figures 3A,D) and vGluT-1-IR (Figure 3B). Even at day 20 after the final stimulation, the average seizure stage in LSKL-treated groups (50 μg, stage 1.88; 100 μg, stage 1.5; and 200 μg, stage 0.25) was significantly lower than that seen in the vehicle control group (stage 4.9; *P* < 0.001, 0.001 and 0.001, respectively; Figure 3F). A similar lower duration of generalized seizures was found after 20 stimulations (*P* < 0.05, 0.001 and 0.001, respectively; Figure 3G). The ADD of rats that received LSKL treatment was also significantly shorter than the saline-treated rats (*P* < 0.001; Figure 3H). After 20 stimulations, the average ADD in the LSKL group (50 μg, 18.38 s; 100 μg, 11.5 s; and 200 μg, 1.25 s) was shorter than that seen in the control group (68.7 s, Figure 3H). In the LSKL 200 μg group, five/eight rats were at stage 0 (no epileptic activity) and showed no AD during kindling progression. The EEGs for each group after 20 stimulations are shown in Figure 3J.

Epileptic susceptibility was assessed by ADT detection after 20 kindling stimulations. In kindled rats treated with saline, ADT decreased by 28 μA, whereas ADT in the 50 μg LSKL group showed a significantly smaller reduction (2.5 μA;* P* < 0.01; Figure 3I). Interestingly, the ADT in the moderate- and high-dose (100 μg and 200 μg) LSKL groups increased (2.5 μA, *P* < 0.001; and 15 μA, *P* < 0.001, respectively; Figure 3I).

**Figure 4:**
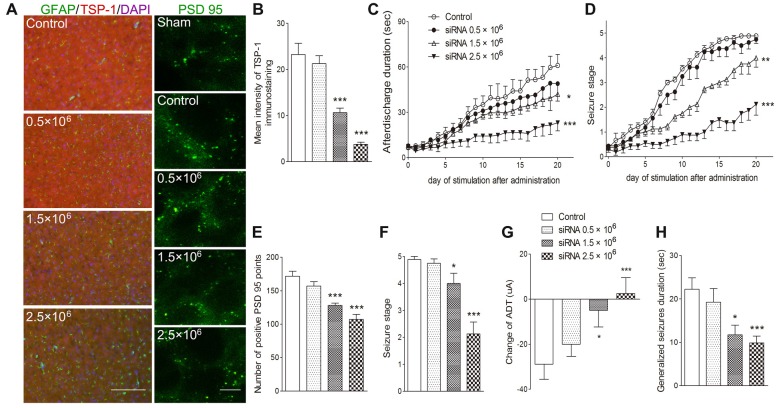
Inhibitory effects of small interfering RNA (siRNA) interference on synapses and kindling progression. **(A)** IR of TSP-1 (red), GFAP (green, bar = 200 μm) and PSD-95 (green, sham group, without kindling; bar = 10 μm) in the cortex, **(B)** mean intensity of TSP-1 immunostaining, **(C)** ADD, **(D)** Racine stage progression of epilepsy, **(E)** number of PSD-95-positive puncta in the field (80 μm × 60 μm), **(F)** seizure stage after 20 stimulations, **(G)** change in the ADT and **(H)** generalized seizure duration in kindling rats (control group, *n* = 9; siRNA groups, *n* = 8–10 per group). Data are shown as mean ± SEM. Asterisks show significant differences from the control group (*P < 0.05; **P < 0.01; ***P < 0.001).

To further verify the role of TSP-1 upregulation during kindling, siRNA interference of TSP-1 expression was performed. When compared with the negative control treatment (control group), administration of 0.5 μg of siRNA failed to significantly affect TSP-1-IR (*P* > 0.05; Figures 4A,B) synaptic number (*P* > 0.05; Figures 4A,E) and epileptic severity or susceptibility (*P* > 0.05; Figures 4C,D,F–H). However, the 2.5 μg dose reduced TSP-1-IR and synaptic number (Figures 4A,B,E) and demonstrated significantly slower progression (*P* < 0.001; Figure 4D), lower average seizure stage (*P* < 0.001; Figure 4F), reduced ADD (*P* < 0.001; Figure 4C), generalized seizure duration (*P* < 0.001; Figure 4H), and epileptic susceptibility (*P* < 0.001; Figure 4G). The 1.5 μg siRNA dose produced effects between the other two doses, indicating a probable dose-response effect (Figures 4A–H). These data indicate that the inhibition of TSP-1 function can reduce synaptic number, delay epileptic progression, and diminish the severity of and the susceptibility to kindling.

### P2 Receptor Antagonism Reduced TSP-1, Synaptic Number, and Epileptic Seizure Activity

**Figure 5:**
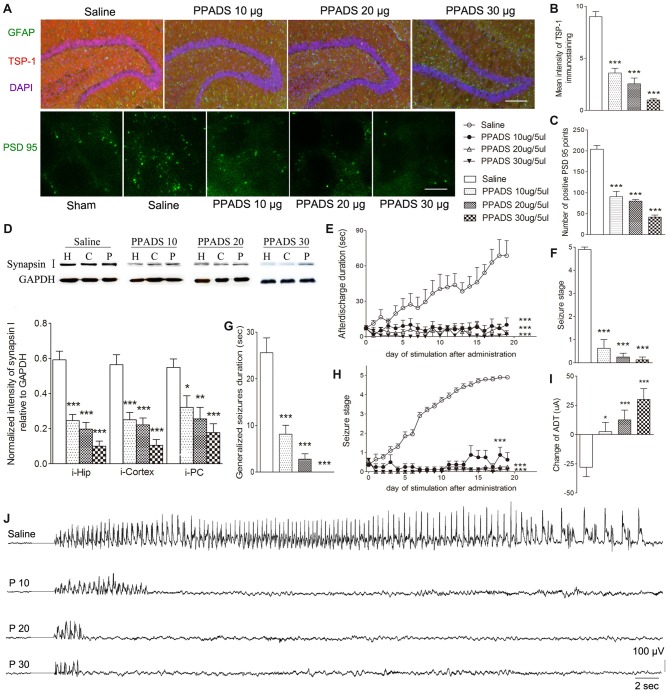
Effect of pyridoxalphosphate-6-azophenyl-2’,4’-disulfonic (PPADS) on TSP-1, synapses and kindling progression. **(A)** IR of TSP-1 (red), GFAP (green, bar = 200 μm), and PSD-95 (green, sham group, without kindling; bar = 10 μm) in the dentate gyrus of the hippocampus, **(B)** mean intensity of TSP-1 immunostaining, **(C)** number of PSD-95-positive puncta in the field (80 μm × 60 μm), **(D)** the ipsilateral IR of synapsin-I after different doses of PPADS (H, hippocampus; P, piriform cortex; C, cortex except PC), **(E)** ADD, **(F)** seizure stage after 19 stimulations, **(G)** duration of generalized seizures, **(H)** Racine stage progression of epilepsy, **(I)** change in the ADT, and **(J)** respective EEGs in kindled rats (saline group, *n* = 10; PPADS groups, *n* = 8–10 per group). The bands were excised from different gels, which were run under the same electrophoresis condition. DAPI, blue. Data are shown as mean ± SEM. Asterisks show significant differences from the control group (*P < 0.05; **P < 0.01; ***P < 0.001).

**Figure 6:**
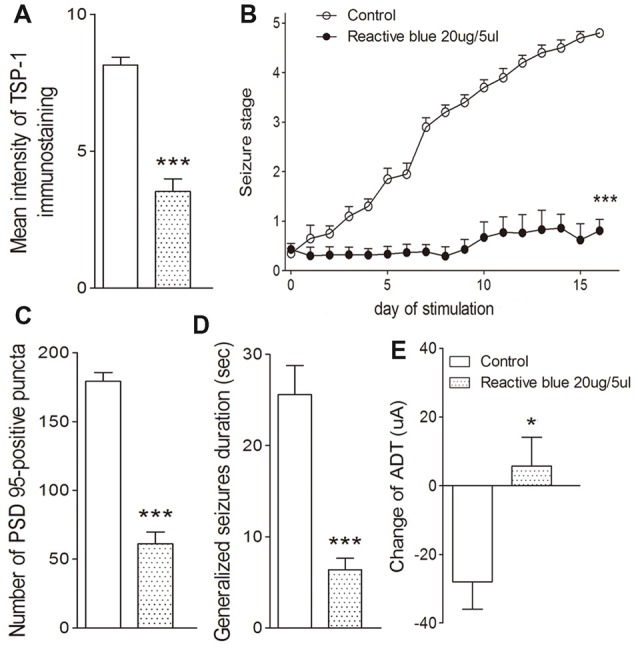
Effect of Reactive Blue 2 on TSP-1, synapses and kindling progression. **(A)** Mean IR of TSP-1, **(B)** Racine stage progression of epilepsy, **(C)** PSD-95 in the dentate gyrus of the hippocampus, **(D)** duration of generalized seizures and **(E)** change in ADT (control group, *n* = 10; Reactive blue group, *n* = 7). Data are shown as mean ± SEM. Asterisks show significant differences from the control group (*P < 0.05; ***P < 0.001).

The broadly-acting P2-type receptor antagonist, PPADS (10, 20, and 30 μg) or the P2Y antagonist, Reactive Blue 2 (20 μg), was administered intracerebroventricularly to evaluate the regulation of TSP-1-IR during kindling by the P2 receptor family. All doses of PPADS significantly reduced the IR of TSP-1 (*P* < 0.001; Figures 5A,B) and the number of synapses (visualized via synapsin-I-IR, Figure 5D), including PSD-95-IR stained excitatory synapses (*P* < 0.001; Figures 5A,C). Epileptic progression was significantly delayed in every PPADS group (*P* < 0.001; Figure 5E). After 19 kindling stimulations, nearly all the rats in the control group demonstrated fully effective kindling; however, six/eight rats in the 30 μg PPADS group were at seizure stage 0 and never demonstrated epileptiform discharges during stimulations. The remaining two rats had progressed only to stage 1. Finally, significantly shorter average ADDs, generalized seizure duration, and significantly attenuated susceptibility and seizure stage were noted in every rat from the PPADS group (*P* < 0.001; Figures 5E–H). Similar inhibition, such as decreased TSP-1/PSD95-IR, attenuated epileptic progression, and reversed ADT, were observed in the Reactive Blue 2 group (Figure 6). The EEGs from every group after 19 stimulations supported this effect (Figure 5J).

## Discussion

Here, we provide the first evidence that increased level of TSP-1 accompanies increased density of synaptic and excitatory synaptic markers in the amygdala-kindling rat epilepsy model. Inhibiting TSP-1 activity with either LSKL or siRNA application led to reduced synaptic/excitatory synaptic formation and seizure activity. Antagonism of P2 receptor family mediated by PPADS or administration of the P2Y antagonist, Reactive Blue 2, attenuated the increased TSP-1 activity and synaptic number and significantly reduced the epileptic activity. Not surprisingly, epilepsy progression and the analyzed biomarkers, as well as increases in the density of synaptic and excitatory synaptic markers and TSP-1-IR, were associated with the anatomical spread of the focal seizures away from the kindled amygdala, through the ipsilateral PC and the hippocampus and then to the remaining ipsilateral cortex and the contralateral regions.

Epilepsy progresses via an “epilepsy network” that requires synaptogenesis to develop (Fidzinski et al., 2015; Heller and Rusakov, 2015); therefore, TSP-1 may be involved in epilepsy-related, as well as developmental, synaptogenesis. In fact, our data demonstrate that TSP-1 increased synchronously with: (1) increased density of synaptic and excitatory synaptic markers in areas with epileptiform activity; (2) increased seizure severity; and (3) increased seizure propagation into networked brain regions (i.e., anatomical progression towards generalized seizures).

Previous reports have shown that along with the progression of kindling with repeated stimuli, the same stimulus intensity produces longer and more widely propagating events that originate from the amygdala, demonstrating that epilepsy requires synaptically networked populations of neurons that generate abnormally synchronized discharges for progression and propagation. This stimulus progressively recruits a larger neural network and generalizes synchronous discharges across the cortex (Hsu, 2007). In kindling-generated focal seizures, the epileptic AD was usually generated focally in the limbic system and then propagated in the generalized seizures (Kanter-Schlifke et al., 2007).

It has been confirmed that the PC is critical to the epileptogenic network, specifically the limbic seizure network (Schwabe et al., 2000, 2004a,b) because of the PC’s extensive connections with other limbic regions. The PC is connected not only to ipsilateral limbic structures but also to contralateral limbic structures such as the contralateral amygdala and PC through the anterior commissure (Schwabe et al., 2004a). Moreover, the PC plays an important role in the maintenance of limbic epileptogenesis and in the development of complex partial seizures (Löscher and Ebert, 1996). The PC may promote generalized seizures directly or indirectly by preferred access (Majak et al., 2004; Schwabe et al., 2004b). Therefore, the PC is regarded as a reasonable target for therapeutic intervention in epilepsy (Schwabe et al., 2000, 2004a,b). In amygdaloid kindling animals, the ipsilateral PC contributes to the pathological changes in the early stages (Ebert and Löscher, 1995; Vessal et al., 2004), further leading to a significant increase in the excitability of the amygdala-PC circuitry. This enhancement plays a critical role in epileptogenesis (McIntyre and Wong, 1985, 1986; Racine et al., 1988; Gean et al., 1989). Based on previous studies, we investigated the changes in IR of proteins such as synapsin-I, PSD-95, vGluT-1, TSP-1 and TGF-β1, especially in the PC. Consistent with previous reports, the IR of synaptic markers, synapsin-I, vGluT-1 and PSD-95, and related proteins, such as TSP-1 and TGF-β1, increased in the ipsilateral PC, beginning at stage 2, and this occurred earlier than in the other cortical regions or hippocampus.

The hippocampus is more sensitive to excitatory input and is considered a “promoter” or “amplifier” of epileptiform discharges (Stringer and Lothman, 1992; Hsu, 2007). Neural network modeling determined that changes in the number of hippocampal synapses, connection strength, and network connectivity can induce epileptiform activity (Netoff et al., 2004). These changes can be visualized in the kainic acid-induced rat epilepsy model; synapsin-I levels increase significantly and bilaterally in the hippocampal CA1 pyramidal cell region (Furtinger et al., 2003). Patients with temporal lobe epilepsy and experimental animal models have demonstrated that abnormal electrical activity of single neurons in the hippocampus can increase excitatory synaptic connections. With excessive excitatory synapses, the normal feedback inhibition in cortical neurons is overwhelmed, and the network takes on epileptic characteristics (Bragin et al., 2002). Therefore, the sensitivity of the hippocampus to this excitatory synaptogenesis causes the amplification effect described above.

In combination with previous studies, this study suggests that the TSP-1/TGF-β1 pathway, regulated by members of the P2 receptor family, is an important component in the progression and propagation of epilepsy. P2 receptors can be activated by ATP, which is a danger signal (Rodrigues et al., 2015) and is released during epileptic seizures. A large elevation in ATP release has been reported in response to pilocarpine-induced status epilepticus (Lietsche et al., 2016), electrical stimulation of the cortex and the Schaffer pathway (Wu and Phillis, 1978; Wieraszko and Seyfried, 1989), and overdischarge of neurons during epilepsy (Henshall and Engel, 2015) *in vivo*. The increased release of ATP was also involved in brain hyperexcitability and increased seizure susceptibility in seizure prone mice (Wieraszko and Seyfried, 1989) and was observed in response to epilepsy induced by high frequency stimulation (Cunha et al., 1996). Moreover, injection of ATP into the brain can exacerbate seizures during status epilepticus (Engel et al., 2012), and the microinjection of ATP analogs into the PC leads to the generation of motor seizures (Knutsen and Murray, 1997). These previous reports indicate that increased ATP contributes to epileptic seizure and may be a crucial trigger in epileptic seizure. Consequently, we speculate that local elevated ATP levels induced by a focal seizure lead to P2 receptor family activation, which increases astrocytic TSP-1 secretion via protein kinase-mediated signaling (Diniz et al., 2012). Increased TSP-1 secretion activates more latent TGF-β1 and, thus, promotes synaptogenesis (Tran and Neary, 2006).

In short, elevated ATP levels form a positive feedback loop in which P2-type receptors, TSP-1 and TGF-β1 increase in activity; this increases the number of excitatory synapses that later promote focal epileptic discharge. In addition, connections to other brain regions are enhanced; therefore, as the intensity of the focal discharge increases, the increased number of synapses on projecting axon terminals from the synchronized neurons will concurrently increase ATP release at their target regions, propagating the discharge until enough populations develop epileptiform activity. At this point, seizures become generalized. Our results support this hypothetical mechanism. We found that the dynamics of TGF-β1 matched those of TSP-1 after kindling. Furthermore, inhibition of either of the two upstream members of the pathway (P2/P2Y receptors or TSP-1) prevented both synaptic increase and seizure activity in terms of both severity and propagation.

Interestingly, neuronal damage was found both in kindled animals, including those with amygdaloid kindling after a period of days to months, and in epileptic patients (DeGiorgio et al., 1992; Wieshmann et al., 1996; Van Landingham et al., 1998; Tuunanen and Pitkänen, 2000). For example, amygdaloid damage and hippocampal damage were observed in about 3% and 10% of patients with temporal lobe epilepsy, respectively (Pitkänen et al., 1998), and a large number of seizures over a lifetime are associated with increased severity of damage in AM (Kälviäinen et al., 1997) or hippocampus (Kälviäinen, 1998). Consequently, activity-dependent neurodegeneration has been regarded as a critical player in epileptogenesis and a target for antiepileptic drugs (Meldrum, 2002).

Amygdaloid kindling is a subconvulsive stimulus. After repeated stimulation, generalized seizures (stages 4 or 5) are eventually evoked (Goddard, 1969). Callahan et al. (1991) reported a decrease in the density of gamma-aminobutyric acid (GABA) neurons several months after 3–5 kindled seizures (Callahan et al., 1991). In our study, we observed changes in the progression (stage 1–5) of amygdaloid kindling before kindled seizures. Unlike the neuronal damage that is observed several months after stage 5 (kindled seizures), an increased number of synapses, especially excitatory synapses, but not neuron loss (data not shown) was observed in different subregions. These results indicate that the contribution of neurons varies during different epileptic periods. In the development of epilepsy, the observed increase in the number of synapses, especially excitatory synapses may contribute to the epileptic network development and in the kindled seizures, and the observed decreases in the number of GABA neurons may promote further overexcitation and neuronal functional defect.

The functional implication of this model supports further testing in clinical situations. The potency with which the P2/P2Y receptor antagonism inhibits both synaptogenesis and seizure activity suggests that they could be strong therapeutic targets, especially in epilepsies that are refractory to other treatments. While TSP-1 inhibition is likely to be effective, the need for intracerebroventricular delivery owing to its small peptide structure makes it the less attractive option. However, TGF-β1 has many effects, so future studies will need to focus on the ability to inhibit this pathway safely and effectively.

## Conclusion

The present study found that increased activity in the TSP-1/TGF-β1 pathway is a critical component in promoting synaptic/excitatory synaptic formation and in increased epileptic seizure progression, in kindling-induced epilepsy. This mechanism might be regulated in part by P2/P2Y purinergic receptors, as demonstrated by the potent regulation of TSP-1, seizure activity, and the density of synaptic markers by PPADS or Reactive Blue 2. Inhibition of this pathway at one or more points could significantly inhibit epileptic activity and/or its spread and might provide novel therapeutic targets for treating epilepsy. Given the broad distribution of P2 receptors in the brain and the relatively broad spectrum of activity of PPADS, further investigation of the specific P2 family receptor subtypes regulating this system is warranted.

## Author Contributions

QW and WZ: study conception and design, data interpretation. YZ and XP: amygdala-kindled rat model preparation, data acquisition, data analysis and interpretation. CW, JZ and XZ: data acquisition and data analysis. HWS: data analysis and interpretation. HLS: study design, critical manuscript revisions, data acquisition and drafting of manuscript. LM: study design, critical manuscript revision and drafting of manuscript.

## Conflict of Interest Statement

The authors declare that the research was conducted in the absence of any commercial or financial relationships that could be construed as a potential conflict of interest.

## Abbreviations

AD: afterdischarge
ADD: afterdischarge duration
ADT: afterdischarge threshold
ANOVA: analysis of variance
GAPDH: glyceraldehyde-3-phosphate dehydrogenase
GFAP: glial fibrillary acidic protein
LSKL: Leu-Ser-Lys-Leu
PPADS: pyridoxalphosphate-6-azophenyl-2’,-4-disulfonic acid
pSmad2/3: phosphorylated Smad2/3
siRNA: small interfering RNA
Smad2/3: small mothers against decapentaplegic 1 and 2
TGF-β1: transforming growth factor beta 1
TSP-1: thrombospondin-1.

## References

[B1] Amakhin D. V. Ergina J. L. Chizhov A. V. Zaitsev A. V. (2016). Synaptic conductances during interictal discharges in pyramidal neurons of rat entorhinal cortex. Front. Cell. Neurosci. 10:233. 10.3389/fncel.2016.00233 27790093 PMC5061778

[B100] Barres B. A. Smith S. J. (2001). Neurobiology. Cholesterol-making or breaking the synapse. Science 294, 1296–1297. 10.1126/science.1066724 11701918

[B2] Bragin A. Mody I. Wilson C. L. Engel J. Jr. (2002). Local generation of fast ripples in epileptic brain. J. Neurosci. 22, 2012–2021. 10.1523/jneurosci.22-05-02012.2002 11880532 PMC6758883

[B3] Burns S. P. Santaniello S. Yaffe R. B. Jouny C. C. Crone N. E. Bergey G. K. (2014). Network dynamics of the brain and influence of the epileptic seizure onset zone. Proc. Natl. Acad. Sci. U S A 111, E5321–E5330. 10.1073/pnas.1401752111 25404339 PMC4267355

[B4] Caciagli L. Bernhardt B. C. Hong S. J. Bernasconi A. Bernasconi N. (2014). Functional network alterations and their structural substrate in drug-resistant epilepsy. Front. Neurosci. 8:411. 10.3389/fnins.2014.00411 25565942 PMC4263093

[B5] Callahan P. M. Paris J. M. Cunningham K. A. Shinnick-Gallagher P. (1991). Decrease of GABA-immunoreactive neurons in the amygdala after electrical kindling in the rat. Brain Res. 555, 335–339. 10.1016/0006-8993(91)90361-x 1933342

[B6] Charvériat M. Naus C. C. Leybaert L. Sáez J. C. Giaume C. (2017). Connexin-dependent neuroglial networking as a new therapeutic target. Front. Cell. Neurosci. 11:174. 10.3389/fncel.2017.00174 28694772 PMC5483454

[B7] Christopherson K. S. Ullian E. M. Stokes C. C. Mullowney C. E. Hell J. W. Agah A.. (2005). Thrombospondins are astrocyte-secreted proteins that promote CNS synaptogenesis. Cell 120, 421–433. 10.1016/j.cell.2004.12.020 15707899

[B8] Codazzi F. Pelizzoni I. Zacchetti D. Grohovaz F. (2015). Iron entry in neurons and astrocytes, a link with synaptic activity. Front. Mol. Neurosci. 8:18. 10.3389/fnmol.2015.00018 26089776 PMC4452822

[B9] Colder B. W. Wilson C. L. Frysinger R. C. Harper R. M. Engel J. Jr. (1996). Interspike intervals during interictal periods in human temporal lobe epilepsy. Brain Res. 719, 96–103. 10.1016/0006-8993(96)00107-2 8782868

[B10] Cunha R. A. Vizi E. S. Ribeiro J. A. Sebastião A. M. (1996). Preferential release of ATP and its extracellular catabolism as a source of adenosine upon high- but not low-frequency stimulation of rat hippocampal slices. J. Neurochem. 67, 2180–2187. 10.1046/j.1471-4159.1996.67052180.x 8863529

[B11] DeGiorgio C. M. Tomiyasu U. Gott P. S. Treiman D. M. (1992). Hippocampal pyramidal cell loss in human status epilepticus. Epilepsia 33, 23–27. 10.1111/j.1528-1157.1992.tb02278.x 1733757

[B12] Diniz L. P. Almeida J. C. Tortelli V. Vargas Lopes C. Setti-Perdigão P. Stipursky J.. (2012). Astrocyte-induced synaptogenesis is mediated by transforming growth factor signaling through modulation of D-serine levels in cerebral cortex neurons. J. Biol. Chem. 287, 41432–41445. 10.1074/jbc.m112.380824 23055518 PMC3510841

[B13] Dubový P. Klusáková I. Hradilová-Svíženská I. Joukal M. Boadas-Vaello P. (2018). Activation of astrocytes and microglial cells and CCL2/CCR2 upregulation in the dorsolateral and ventrolateral nuclei of periaqueductal gray and rostral ventromedial medulla following different types of sciatic nerve injury. Front. Cell. Neurosci. 12:40. 10.3389/fncel.2018.00040 29515373 PMC5825898

[B14] Ebert U. Löscher W. (1995). Strong induction of c-fos in the piriform cortex during focal seizures evoked from different limbic brain sites. Brain Res. 671, 338–344. 10.1016/0006-8993(94)01401-3 7743227

[B15] Engel T. Gómez-Villafuertes R. Tanaka K. Mesuret G. Sanz-Rodriguez A. Garcia-Huerta P.. (2012). Seizure suppression and neuroprotection by targeting the purinergic P2X7 receptor during status epilepticus in mice. FASEB J. 26, 1616–1628. 10.1096/fj.11-196089 22198387

[B16] Feldt Muldoon S. Soltesz I. Cossart R. (2013). Spatially clustered neuronal assemblies comprise the microstructure of synchrony in chronically epileptic networks. Proc. Natl. Acad. Sci. U S A 110, 3567–3572. 10.1073/pnas.1216958110 23401510 PMC3587208

[B17] Fidzinski P. Korotkova T. Heidenreich M. Maier N. Schuetze S. Kobler O.. (2015). KCNQ5 K^(+)^ channels control hippocampal synaptic inhibition and fast network oscillations. Nat. Commun. 6:6254. 10.1038/ncomms7254 25649132

[B18] Furtinger S. Bettler B. Sperk G. (2003). Altered expression of GABA_B_ receptors in the hippocampus after kainic-acid-induced seizures in rats. Mol. Brain Res. 113, 107–115. 10.1016/s0169-328x(03)00097-4 12750012

[B19] Garcia-Ramos C. Bobholz S. Dabbs K. Hermann B. Joutsa J. Rinne J. O.. (2017). Brain structure and organization five decades after childhood onset epilepsy. Hum. Brain Mapp. 38, 3289–3299. 10.1002/hbm.23593 28370719 PMC5479770

[B20] Gean P. W. Shinnick Gallagher P. Anderson A. C. (1989). Spontaneous epileptiform activity and alteration of GABA- and of NMDA-mediated neurotransmission in amygdala neurons kindled *in vivo*. Brain Res. 494, 177–181. 10.1016/0006-8993(89)90160-1 2670063

[B21] Goddard G. V. (1969). Analysis of avoidance conditioning following cholinergic stimulation of amygdala in rats. J. Comp. Physiol. Psychol. 68, 1–18. 10.1037/h0027504 5784697

[B22] Haneef Z. Chiang S. Yeh H. J. Engel J. Stern J. M. (2015). Functional connectivity homogeneity correlates with duration of temporal lobe epilepsy. Epilepsy Behav. 46, 227–233. 10.1016/j.yebeh.2015.01.025 25873437 PMC4458387

[B23] Heller J. P. Rusakov D. A. (2015). Morphological plasticity of astroglia: understanding synaptic microenvironment. Glia 63, 2133–2151. 10.1002/glia.22821 25782611 PMC4737250

[B24] Henshall D. C. Engel T. (2015). P2X purinoceptors as a link between hyperexcitability and neuroinflammation in status epilepticus. Epilepsy Behav. 49, 8–12. 10.1016/j.yebeh.2015.02.031 25843343

[B25] Hsu D. (2007). The dentate gyrus as a filter or gate, a look back and a look ahead. Prog. Brain Res. 163, 601–613. 10.1016/s0079-6123(07)63032-5 17765740

[B26] Kälviäinen R. (1998). Tiagabine: a new therapeutic option for people with intellectual disability and partial epilepsy. J. Intellect. Disabil. Res. 42, 63–67. 10030435

[B27] Kälviäinen R. Salmenperä T. Partanen K. Vainio P. Riekkinen P. Pitkänen A. (1997). MRI volumetry and T2 relaxometry of the amygdala in newly diagnosed and chronic temporal lobe epilepsy. Epilepsy Res. 28, 39–50. 10.1016/s0920-1211(97)00029-6 9255598

[B28] Kanter-Schlifke I. Georgievska B. Kirik D. Kokaia M. (2007). Seizure suppression by GDNF gene therapy in animal models of epilepsy. Mol. Ther. 15, 1106–1113. 10.1038/sj.mt.6300148 17387333

[B30] Knutsen L. J. S. Murray T. (1997). “Adenosine and ATP in epilepsy,” in Purinergic Approaches in Experimental Therapeutics, eds Jakobson K. A. Jarvis M. F. (New York, NY: Wiley-Liss), 432–447.

[B31] Kros L. Lindeman S. Eelkman Rooda O. H. J. Murugesan P. Bina L. Bosman L. W. J.. (2017). Synchronicity and rhythmicity of purkinje cell firing during generalized spike-and-wave discharges in a natural mouse model of absence epilepsy. Front. Cell. Neurosci. 11:346. 10.3389/fncel.2017.00346 29163057 PMC5671558

[B32] Lietsche J. Imran I. Klein J. (2016). Extracellular levels of ATP and acetylcholine during lithium-pilocarpine induced status epilepticus in rats. Neurosci. Lett. 611, 69–73. 10.1016/j.neulet.2015.11.028 26610905

[B33] Löscher W. Ebert U. (1996). The role of the piriform cortex in kindling. Prog. Neurobiol. 50, 427–481. 10.1016/s0301-0082(96)00036-6 9015822

[B34] Lupica C. R. Hu Y. Devinsky O. Hoffman A. F. (2017). Cannabinoids as hippocampal network administrators. Neuropharmacology 124, 25–37. 10.1016/j.neuropharm.2017.04.003 28392266

[B29] Majak K. Rönkkö S. Kemppainen S. Pitkänen A. (2004). Projections from the amygdaloid complex to the piriform cortex: a PHA-L study in the rats. J. Comp. Neurol. 476, 414–428. 10.1002/cne.20233 15282713

[B36] McIntyre D. C. Wong R. K. (1985). Modification of local neuronal interactions by amygdala kindling examined *in vitro*. Exp. Neurol. 88, 529–537. 10.1016/0014-4886(85)90068-8 3996508

[B37] McIntyre D. C. Wong R. K. (1986). Cellular and synaptic properties of amygdala-kindled pyriform cortex *in vitro*. J. Neurophysiol. 55, 1295–1307. 10.1152/jn.1986.55.6.1295 3016209

[B38] Meldrum B. S. (2002). Implications for neuroprotective treatments. Prog. Brain Res. 135, 487–495. 10.1016/s0079-6123(02)35046-5 12143367

[B39] Milton M. Smith P. D. (2018). It’s all about timing: the involvement of Kir4.1 channel regulation in acute ischemic stroke pathology. Front. Cell. Neurosci. 12:36. 10.3389/fncel.2018.00036 29503609 PMC5820340

[B40] Murphy-Royal C. Dupuis J. P. Varela J. A. Panatier A. Pinson B. Baufreton J.. (2015). Surface diffusion of astrocytic glutamate transporters shapes synaptic transmission. Nat. Neurosci. 8, 219–226. 10.1038/nn.3901 25581361

[B41] Netoff T. I. Clewley R. Arno S. Keck T. White J. A. (2004). Epilepsy in small-world networks. J. Neurosci. 24, 8075–8083. 10.1523/JNEUROSCI.1509-04.2004 15371508 PMC6729784

[B42] Pfieger F. W. Barres B. A. (1997). Synaptic efficacy enhanced by glial cells *in vitro*. Science 277, 1684–1687. 10.1126/science.277.5332.1684 9287225

[B43] Pitkänen A. Tuunanen J. Kälviäinen R. Partanen K. Salemenperá T. (1998). Amygdala damage in experimental and human temporal lobe epilepsy. Epilepsy Res. 32, 233–253. 10.1016/s0920-1211(98)00055-2 9761324

[B44] Racine R. J. Mosher M. Kairiss E. W. (1988). The role of the pyriform cortex in the generation of interictal spikes in the kindled preparation. Brain Res. 454, 251–263. 10.1016/0006-8993(88)90825-6 3409009

[B45] Ribeiro S. M. Poczatek M. Schultz-Cherry S. Villain M. Murphy-Ullrich J. E. (1999). The activation sequence of thrombospondin-1 interacts with the latency-associated peptide to regulate activation of latent transforming growth factor-β. J. Biol. Chem. 274, 13586–13593. 10.1074/jbc.274.19.13586 10224129

[B46] Rodrigues R. J. Tomé A. R. Cunha R. A. (2015). ATP as a multi-target danger signal in the brain. Front. Neurosci. 9:148. 10.3389/fnins.2015.00148 25972780 PMC4412015

[B47] Sánchez-Ramón S. Faure F. (2017). The thymus/neocortex hypothesis of the brain: a cell basis for recognition and instruction of self. Front. Cell. Neurosci. 11:340. 10.3389/fncel.2017.00340 29163052 PMC5663735

[B49] Schwabe K. Ebert U. Löscher W. (2000). Bilateral lesions of the central but not anterior or posterior parts of the piriform cortex retard amygdala kindling in rats. Neuroscience 101, 513–521. 10.1016/s0306-4522(00)00407-3 11113300

[B50] Schwabe K. Ebert U. Löscher W. (2004a). The central piriform cortex: anatomical connections and anticonvulsant effect of GABA elevation in the kindling model. Neuroscience 126, 727–741. 10.1016/j.neuroscience.2004.04.022 15183521

[B51] Schwabe K. Ebert U. Löscher W. (2004b). Bilateral microinjections of vigabatrin in the central piriform cortex retard amygdala kindling in rats. Neuroscience 129, 425–429. 10.1016/j.neuroscience.2004.08.013 15501599

[B52] Stringer J. L. Lothman E. W. (1992). Bilateral maximal dentate activation is critical for the appearance of an afterdischarge in the dentate gyrus. Neuroscience 46, 309–314. 10.1016/0306-4522(92)90053-5 1542409

[B53] Sun H. L. Deng D. P. Pan X. H. Wang C. Y. Zhang X. L. Chen X. M.. (2016). A sub-threshold dose of pilocarpine increases glutamine synthetase in reactive astrocytes and enhances the progression of amygdaloid-kindling epilepsy in rats. Neuroreport 27, 213–219. 10.1097/WNR.0000000000000511 26684398

[B54] Sun H. L. Zhang S. H. Zhong K. Xu Z. H. Feng B. Yu J.. (2013). A transient upregulation of glutamine synthetase in the dentate gyrus is involved in epileptogenesis induced by amygdala kindling in the rat. PLoS One 8:e66885. 10.1371/journal.pone.0066885 23825580 PMC3688959

[B55] Sun H. L. Zhu W. Zhang Y. R. Pan X. H. Zhang J. R. Chen X. M.. (2017). Altered glutamate metabolism contributes to antiepileptogenic effects in the progression from focal seizure to generalized seizure by low-frequency stimulation in the ventral hippocampus. Epilepsy Behav. 68, 1–7. 10.1016/j.yebeh.2016.09.009 28109982

[B57] Tran M. D. Furones-Alonso O. Sanchez-Molano J. Bramlett H. M. (2012). Trauma-induced expression of astrocyte thrombospondin-1 is regulated by P2 receptors coupled to protein kinase cascades. Neuroreport 23, 721–726. 10.1097/WNR.0b013e32835688fe 22776902

[B56] Tran M. D. Neary J. T. (2006). Purinergic signaling induces thrombospondin-1 expression in astrocytes. Proc. Natl. Acad. Sci. U S A 103, 9321–9326. 10.1073/pnas.0603146103 16754856 PMC1482608

[B58] Tuunanen J. Pitkänen A. (2000). Do seizures cause neuronal damage in rat amygdala kindling? Epilepsy Res. 39, 171–176. 10.1016/s0920-1211(99)00123-0 10759304

[B59] Ullian E. M. Sappersyein S. K. Christopherson K. S. Barres B. A. (2001). Control of synapse number by glia. Science 291, 657–661. 10.1126/science.291.5504.657 11158678

[B60] van Deijk A. F. Camargo N. Timmerman J. Heistek T. Brouwers J. F. Mogavero F.. (2017). Astrocyte lipid metabolism is critical for synapse development and function *in vivo*. Glia 65, 670–682. 10.1002/glia.23120 28168742

[B61] Van Landingham K. E. Heinz E. R. Cavazos J. E. Lewis D. V. (1998). Magnetic resonance imaging evidence of hippocampal injury after prolonged focal febrile convulsions. Ann. Neurol. 43, 413–426. 10.1002/ana.410430403 9546321

[B35] Vessal M. Dugani C. B. Solomon D. A. Burnham W. M. Ivy G. O. (2004). Astrocytic proliferation in the piriform cortex of amygdalakindled subjects: a quantitative study in partial versus fully kindled brains. Brain Res. 1022, 47–53. 10.1016/j.brainres.2004.06.071 15353212

[B62] Wieraszko A. Seyfried T. N. (1989). Increased amount of extracellular ATP in stimulated hippocampal slices of seizure prone mice. Neurosci. Lett. 106, 287–293. 10.1016/0304-3940(89)90178-x 2532311

[B63] Wieshmann U. C. Free S. L. Everitt A. D. Bartlett P. A. Barker G. J. Tofts P. S.. (1996). Magnetic resonance imaging in epilepsy with a fast FLAIR sequence. J. Neurol. Neurosurg. Psychiatry. 61, 357–361. 10.1136/jnnp.61.4.357 8890773 PMC486575

[B64] Wu P. H. Phillis J. W. (1978). Distribution and release of adenosine triphosphate in rat brain. Neurochem. Res. 3, 563–571. 10.1007/bf00963759 370672

[B65] Xiao F. Lei D. An D. Li L. Chen S. Chen F.. (2015). Functional brain connectome and sensorimotor networks in rolandic epilepsy. Epilepsy Res. 113, 113–125. 10.1016/j.eplepsyres.2015.03.015 25986198

[B66] Xu Y. Qiu S. Wang J. Liu Z. Zhang R. Li S.. (2014). Disrupted topological properties of brain white matter networks in left temporal lobe epilepsy, a diffusion tensor imaging study. Neuroscience 279, 155–167. 10.1016/j.neuroscience.2014.08.040 25194789

